# Design of an Integrated Optics Sensor Structure Based on Diamond Waveguide for Hemoglobin Property Detection

**DOI:** 10.3390/ma12010175

**Published:** 2019-01-07

**Authors:** Przemysław Struk

**Affiliations:** Department of Optoelectronics, Faculty of Electrical Engineering, Silesian University of Technology, 44-100 Gliwice, Poland; Przemyslaw.Struk@polsl.pl; Tel.: +48-32-237-2182

**Keywords:** integrated optics structures, diamond waveguide layer, hemoglobin biosensor

## Abstract

This manuscript presents a theoretical analysis of a diamond-based integrated optics structure for applications in biosensors. The geometrical, optical, and sensitivity properties of an integrated optical structure were theoretically analyzed and optimized for biosensor applications. The analysis focused on determining the waveguide properties, including the effective refractive index *N_eff_* as a function of refractive index *n_w_* and thickness *d_w_* of waveguide layer, refractive index of the hemoglobin cover layer *n_cH_* and substrate layer *n_s_*, homogeneous sensitivity *dN_eff_/dn_cH_*, and modal field distribution of guided waveguide modes. The analysis was completed for two types of waveguide layer materials: undoped or boron-doped diamond films with or without the hemoglobin cover layer. The presented experimental results form a base for developing biosensor structures based on integrated optics for determining the properties of hemoglobin.

## 1. Introduction

Modern medical care requires fast, accurate, possibly painless, and minimally invasive methods for patient diagnosis that can be used to quickly diagnose diseases in the human body. One basic diagnosis method for verifying a patient’s health is research into blood morphology [1]. The key component of blood is hemoglobin; its level and oxidation have a direct impact on the proper function of living organisms [2]. Hemoglobin is directly responsible for oxygen transport between pulmonary alveolus and body cells that use oxygen for metabolic processes [3]. From this perspective, it is important to develop sensor structures which could be used to determine the physical properties of blood, particularly hemoglobin level and oxidation. An interesting group of sensors which could be applied for this purpose are fiber-based and integrated optics-based sensors structures [4,5,6]. The advantages of integrated optics sensors are their high scale of integration, high accuracy, and low cost. The operating principle of an integrated optics structure for sensors and biosensors applications is mostly based on exploiting the evanescent field of a guided waveguide mode or based on exploiting the grating couplers [7,8,9,10,11,12,13,14,15]. The evanescent field of a guided waveguide mode penetrates the cover layer and the substrate layer [7,8,9,10,11,12,13,14,15]. Changes in the optical properties (e.g., refractive index or extinction coefficient) and geometric properties (e.g., thickness) of the cover layer affects on the effective refractive index or attenuation coefficient of light into waveguide structure. This produces changes in the propagation conditions for waveguide modes which could be detected.

The most important issue surrounding the development of integrated optics structures is the choice of a suitable material that can act as a waveguide layer in a planar waveguide structure. An interesting group of materials that can be applied as waveguide layer is diamond material [16,17,18]. Diamond thin films are optically transparent in the visible and near infrared regions with refractive index *n* ranging from 2.1 to 2.3 (depending of the deposition technology and doping elements) [19,20,21]. The advantages of using diamond materials are their high mechanical durability, high chemical stability, and high chemical resistance [19,20,21]. In addition, diamond films are biocompatible with tissues [22]. It should be noted that the fabrication of the diamond-based waveguide layer with appropriate optical and geometrical properties is a challenge for deposition technology. However, the experimental results presented by Sobaszek et al. and Ficek et al. [19,20] have shown a diamond layer with high optical transmission and high refractive index which could be modulated by deposition technology and doping materials. According to results presented by Sobaszek et al. [19], the diamond layer could be fabricated with a thickness of several hundred nanometers with a low surface roughness at the level of few nm.

This manuscript presents a theoretical analysis of the waveguide properties of a diamond-based integrated optics sensor structure with a hemoglobin cover layer. The presented theoretical analysis focuses on optimizing the optical and geometric properties of integrated optic sensor structures for determining the physical properties of hemoglobin. The presented theoretical analysis provides a basis for fabricating a sensor structure with optimized optical and geometrical properties that yield the highest sensitivities. 

## 2. Methods

**Figure 1:**
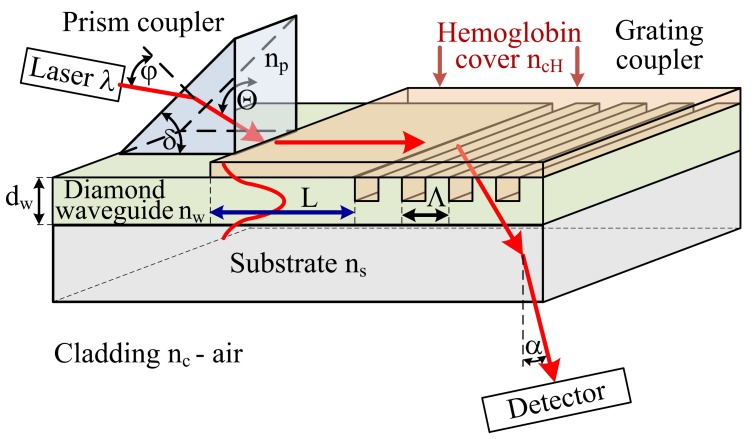
Scheme of the hemoglobin sensor structure, *n_p_*—refractive index of prism, *n_w_*—refractive index of waveguide layer, *n_s_*—refractive index of substrate, *n_cH_*—refractive index of hemoglobin cover layer, *n_c_*—refractive index of cladding, *d_w_*—thickness of the waveguide layer, *ϕ*—angle of incidence of the light beam from laser upon the prism, *Θ*—angle of incidence of the light beam upon the base of the prism, *δ*—breaking angle of the prism, *L*—length of the waveguide with hemoglobin cover layer, *α*—angle of the uncoupling light beam by grating coupler, Λ—spatial period of the grating coupler, *λ*—wavelength of light.

The concept of the hemoglobin sensor structure is presented in Figure 1 The sensor is composed of three sections: prism coupler, planar waveguide with length *L* and grating coupler with spatial period Λ. In the case of hemoglobin, two parameters are most interesting for the determination of patient health: hemoglobin level and hemoglobin oxidation. The hemoglobin level and hemoglobin oxidation correspond to the optical properties such as: refractive index *n* (real part) and imaginary part of refractive index *k* [23,24,25]. These two parameters could be measure by proposed hemoglobin sensor. The theoretical analysis of proposed integrated optics-based hemoglobin sensor is presented below. Several physical, geometric, and optical properties of a planar waveguide structure (e.g., thickness *d_w_*; refractive indices of the waveguide layer *n_w_* (undoped or boron-doped diamond), substrate *n_s_* (quartz), cladding *n_c_* (air) and cover layer *n_ch_* (hemoglobin)) have an impact on various waveguide properties (e.g., effective refractive index *N_eff_*, number of guided waveguide modes, homogeneous sensitivity *dN_eff_/dn_cH_*, and modal field distribution). Theoretical analysis was conducted for two types of waveguide layer materials: undoped diamond film (NDD) or boron-doped diamond film (BDD). The NDD film has a refractive index of *n*~2.3 (560 nm) [21]. However, the refractive index could be decreased to *n*~2.19 (560 nm) by doping the diamond layer by boron [21]. Changes in the refractive index of the waveguide layer allows one to modify the waveguide properties of an integrated optics sensor structure, as shown below. The analysis was completed for an integrated optics structure with and without a hemoglobin cover layer and for grating coupler. The scheme of the analyzed structures is presented in Figure 1.

The prism coupler is responsible for coupling light from a laser into a planar waveguide. The light could be coupled into a waveguide if components of the wave vector along the way of propagation in the prism and in the waveguide layer have the same value, and correspond to one of the possible propagation components in the waveguide structure [7,10,12]:

(1) $$\frac{2\pi }{\lambda_{0}}n_{p}\sin\Theta \;=\;\beta_{w}\;=\;\frac{2\pi }{\lambda_{0}}N_{eff}$$

where: *n_p_*—refractive index of prism, *Θ*—angle of incidence of the light beam upon the base of the prism, *β*_w_—propagation constant in the waveguide layer, *N_eff_*—effective refractive index.

The grating coupler is responsible for uncoupling the light from waveguide to cladding. The mathematical condition of uncoupling light from waveguide by grating coupler is following [9,10]:

(2) $$\beta_{c}\sin\left(\alpha \right)\;=\;\beta_{w}+\frac{m_{o}2\pi }{\Lambda }$$

where: *β_c_*—propagation constant in the environment; *β_w_*—propagation constant in the waveguide structure, Λ—space period of the grating, *m_o_*—diffraction order.

It should be noted that the grating coupler acts as a sensor for the determination of the refractive index of the cover layer and hence, the refractive index of hemoglobin. Changes of refractive index in the cover layer *n_cH_* and hence, causing changes in effective refractive index *N_eff_* have influence of the uncoupling angle *α* (measured by detector) [7,12]:

(3) $$\alpha \;=\;\arcsin\left[\frac{1}{n_{c}}\left(N_{eff}-\frac{m_{o}\lambda }{\Lambda }\right)\right]$$

The planar waveguide with length *L* is responsible for the determination of absorption coefficient (imaginary part of refractive index *k*) of hemoglobin cover layer by evanescence field of guided mode. The evanescence field of guided mode penetrate the hemoglobin cover layer and hence is attenuated, the output power of light is detected by detector. The highest changes of imaginary part of refractive index for oxidized and non-oxidized hemoglobin is for wavelength: first maximum 436 nm and second 560 nm [25]. 

The analysis presented below were carried out for wavelength 560 nm, for which the refractive indices of each layer in analyzed structure, especially refractive index of hemoglobin (as a function concentration) are well known with high accuracy Table 1.

The effective refractive index *N_eff_* for a guided mode depends on the optical properties and geometry of the planar waveguide structure, including the refractive index of the substrate *n_s_*, refractive index of the waveguide layer *n_w_*, its thickness *d_w_*, refractive index of the cover layer *n_cH_*, wavelength *λ*, and polarization of light. The modal equations for TE—Transverse Electric or TM—Transverse Magnetic polarization are following [26]:

Transverse electric mode (TE)

(4) $$k_{o}\times d_{w}\sqrt{n_{w}^{2}-N_{effTEm}^{2}}\;=\;arctg\left(\sqrt{\frac{N_{effTEm}^{2}-n_{cH}^{2}}{n_{w}^{2}-N_{effTEm}^{2}}}\right)+arctg\left(\sqrt{\frac{N_{effTEm}^{2}-n_{s}^{2}}{n_{w}^{2}-N_{effTEm}^{2}}}\right)+m\pi \;$$

Transverse magnetic mode (TM)

(5) $$k_{o}\times d_{w}\sqrt{n_{w}^{2}-N_{effTMm}^{2}}\;=\;arctg\left(\sqrt{\frac{N_{effTMm}^{2}-n_{cH}^{2}}{n_{w}^{2}-N_{effTMm}^{2}}}\times {\left(\frac{n_{w}}{n_{cH}}\right)}^{2}\right)+arctg\left(\sqrt{\frac{N_{effTMm}^{2}-n_{s}^{2}}{n_{w}^{2}-N_{\;effTMm}^{2}}}\times {\left(\frac{n_{w}}{n_{s}}\right)}^{2}\right)+m\pi \;$$

where: *k_o_ = 2π/λ*—wave number, *d_w_*—waveguide layer thickness, *n_w_*—refractive index of waveguide layer, *n_cH_*—refractive index of cover layer (Figure 1), *n_s_*—refractive index of substrate, *N_effTEm_*—effective refractive index of m-order mode for TE polarization, *N_effTMm_*—effective refractive index of m-order mode for TM polarization, *m*—waveguide mode order (1, 2, 3, …).

The homogeneous sensitivity with respect of changes refractive index of cover layer *n_cH_* (hemoglobin) as a function of the waveguide layer thickness *d_w_* could be calculated assuming small changes of refractive index of cover layer [27]:

(6) $$S\;=\;\frac{dN_{eff}}{dn_{cH}}\;\approx \frac{\Delta N_{eff}}{\Delta n_{cH}}$$

**Figure 2:**
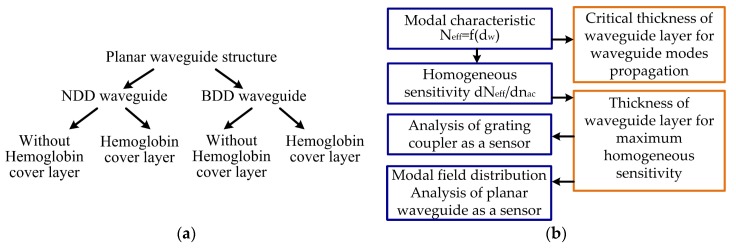
Scheme of the theoretical analysis: (**a**) types of diamond-based planar waveguide structures, (**b**) analysis steps.

A schematic view of the theoretical analysis of the integrated optics structure is presented in Figure 2a, and analysis steps are shown in Figure 2b. The refractive index for each layer (NDD or BDD waveguide layer *n_w_*, quartz substrate *n_s_*, and hemoglobin cover layer *n_cH_*) in the modeled planar waveguide structure is described in Table 1. 

The numerical analysis of the effective refractive index *N_eff_*, modal field distribution and waveguide modes propagation were obtained by OptiFDTD 32 bit software (Optiwave, Ottawa, ON, Canada). 

## 3. Calculation Results

The first section of numerical analysis focuses on determining the effective refractive index *N_eff_* as a function of waveguide thickness *d_w_*. The following section focuses on determining the homogeneous sensitivity *dN_eff_/dn_cH_* (i.e., changes in *N_eff_* with respect of changes in the refractive index of the cover layer *n_cH_*) as a function of the waveguide layer thickness *d_w_*. The final section focuses on numerically determining the modal field distribution for guided waveguide modes as well as propagation of modes in diamond–based planar waveguide structure with hemoglobin cover.

### 3.1. Modal Characteristics of the Planar Waveguide Structure

**Figure 3:**
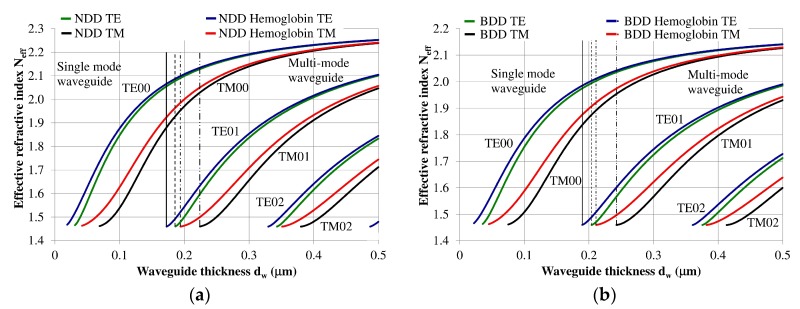
Modal characteristics of waveguide structure based on: (**a**) undoped diamond waveguide layer, (**b**) boron-doped diamond waveguide layer. The following vertical lines indicate the border between single or multi-mode propagation: solid line: waveguide structure with hemoglobin cover layer (TE polarization), dashed line: waveguide structure without hemoglobin cover (TE polarization), dashed line with a single dot: waveguide structure with hemoglobin cover layer (TM polarization), dashed line with a double dot: waveguide structure without hemoglobin cover layer (TM polarization).

The planar waveguide structure and the optical and geometrical properties of each layer can be described using an effective refractive index *N_eff_* [7,12]. The effective refractive index as a function of waveguide layer thickness *N_eff_ = f*(*d_w_*) for the NDD-based or BDD-based planar waveguide structures are shown in Figure 3a,b, respectively. Vertical lines in these figures define thresholds corresponding to the transition from single-mode to multi-mode propagation. If the waveguide thickness is less than the marked lines, then the structure will only support a single propagating mode. Analogously, if the waveguide layer thickness is greater than the marked vertical lines, then the structure will support multi-mode propagation. 

The NDD-based waveguide has a higher effective refractive index for waveguide modes compared to the DBB-based waveguide for a given waveguide layer thickness. In addition, the critical thickness of the waveguide layer for which a mode can be guided is lower for the NDD-based waveguide compared to the BDD-based waveguide. Detailed information regarding the critical thickness of the waveguide layer is presented in Table 2.

### 3.2. The Homogeneous Sensitivity dN_eff_/dn_cH_

**Figure 4:**
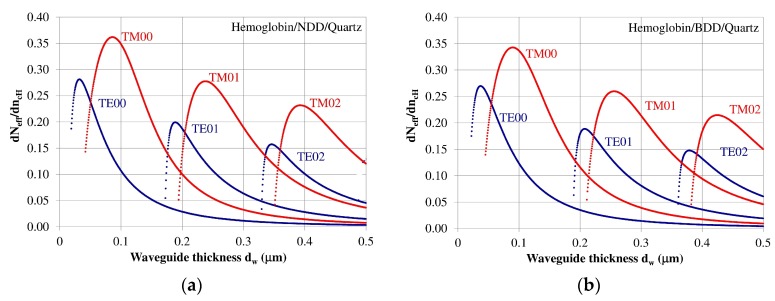
Homogeneous sensitivity *dN_eff_*/*dn_cH_* for the (**a**) undoped diamond (NDD) and (**b**) boron-doped diamond (BDD) waveguide structures.

The homogeneous sensitivity *dN_eff_/dn_cH_*—sensitivity with respect to changes in refractive index of cover layer *n_cH_* (hemoglobin) as a function of the waveguide layer thickness *d_w_* is dependent on the refractive index distribution in the planar waveguide, polarization of light, waveguide mode order, and the thickness of the waveguide layer. The homogeneous sensitivity *dN_eff_/dn_cH_* was analyzed for guided waveguide modes in the NDD or BDD planar waveguide structures with a hemoglobin cover layer. The structure based on an NDD waveguide layer exhibited the highest homogeneous sensitivity *dN_eff_/dn_cH_*, as this structure has the highest refractive index contrast between the waveguide, substrate, and cover layer. The homogeneous sensitivity in the of BDD-based waveguide structure is a bit lower compared to the NDD-based structure due to its lower contrast of refractive index between the waveguide and the surrounding layers. The homogeneous sensitivity *dN_eff_/dn_cH_* for the NDD and BDD-based waveguide structures is presented in Figure 4a,b, respectively.

The homogeneous sensitivity *dN_eff_/dn_cH_* is highest for zero order modes TE00 and TM00 and it decreased for modes TE01, TM01, TE02, and TM02. The homogeneous sensitivity also depends on the polarization and is higher for Transverse Magnetic (TM) modes, and lower for Transverse Electric (TE) modes. The thicknesses of the waveguide layer corresponding to maximum homogeneous sensitivity for modes TE00, TE01, TE02, TM00, TM01, and TM02 are presented in Table 3. The results from the theoretical analysis presented above show that a maximum homogeneous sensitivity is observed when the waveguide layer thickness is somewhat higher than the critical thickness that allows a guided mode to propagate in the planar waveguide structure. 

The theoretical analysis presented shows that an undoped diamond-based (NDD) planar waveguide structure has higher homogeneous sensitivity than a boron-doped diamond-based waveguide. The undoped diamond layer has higher refractive index than the boron-doped diamond layer; hence, it has higher values of effective refractive indices for individual waveguide modes for a specified waveguide layer thickness. In addition, the critical thickness of the waveguide layer based on undoped diamond is smaller compared to the boron-doped diamond layer. 

The expected changes of the angle Δ*α* of uncoupling light by grating coupler (Figure 1) as a function of hemoglobin concentration in distilled water 0–26 g/dL was estimated by Equation (3). The refractive index of hemoglobin in distilled water is presented in Table 1. The analyses were completed for the waveguide thickness corresponding to maximum homogenous sensitivity and assuming the spatial period of the grating Λ = 600 nm and first diffraction order *m* = 1; the results are presented in Table 3. 

The calculated changes of the angle ∆*α* for uncoupled light beam by grating coupler as a function of hemoglobin concentration (corresponding to hemoglobin refractive index) is at the level of ∆*α* = 0.48°–1.28° depending on the waveguide modes. The highest ∆*α* is for zero modes in a single waveguide mode; the ∆*α* decrease for higher order mode in multimode waveguide.

### 3.3. Modal Field Distribution

**Figure 5:**
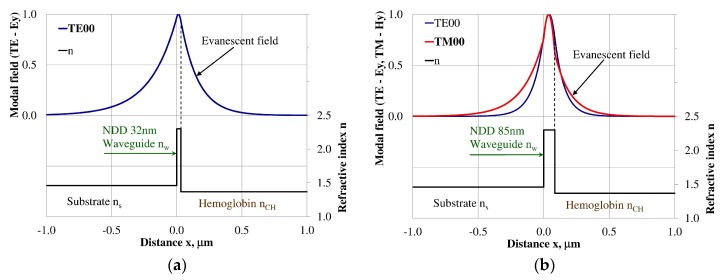
Modal field distribution in the single-mode NDD-based waveguide: (**a**) TE00 for maximum *dN_eff_*/*dn_cH_*, and (**b**) TM00 for maximum *dN_eff_*/*dn_cH_*.

**Figure 6:**
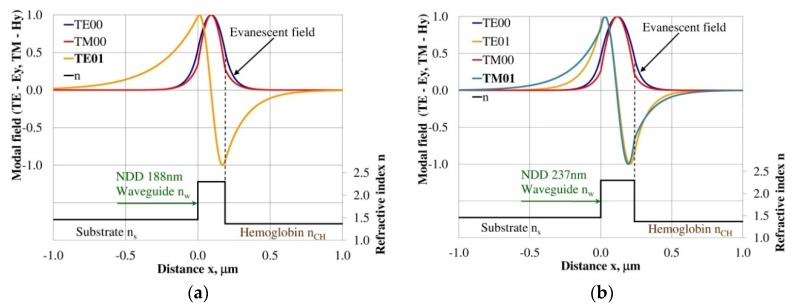
Modal field distribution in the multi-mode NDD-based waveguide: (**a**) TE01 for maximum *dN_eff_/dn_cH_* and (**b**) TM01 for maximum *dN_eff_/dn_cH_*.

**Figure 7:**
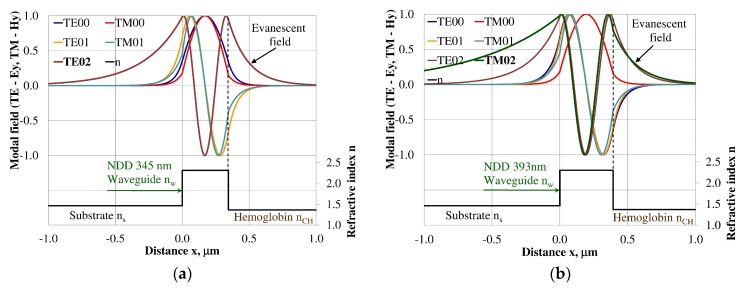
Modal field distribution in the multi-mode NDD-based waveguide: (**a**) TE02 for maximum *dN_eff_/dn_cH_*, and (**b**) TM02 for maximum *dN_eff_/dn_cH_*.

**Figure 8:**
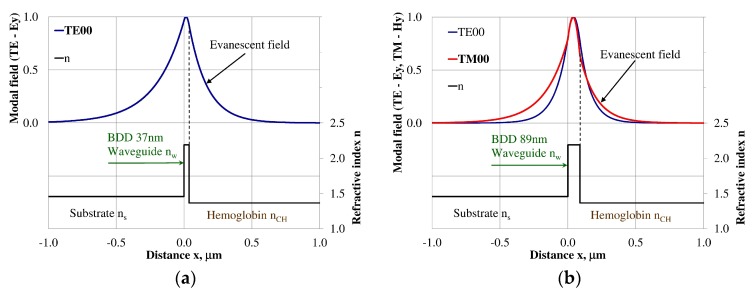
The modal field distribution in the single-mode BDD-based waveguide: (**a**) TE00 for maximum *dN_eff_/dn_cH_*, and (**b**) TM00 for maximum *dN_eff_/dn_cH_*.

**Figure 9:**
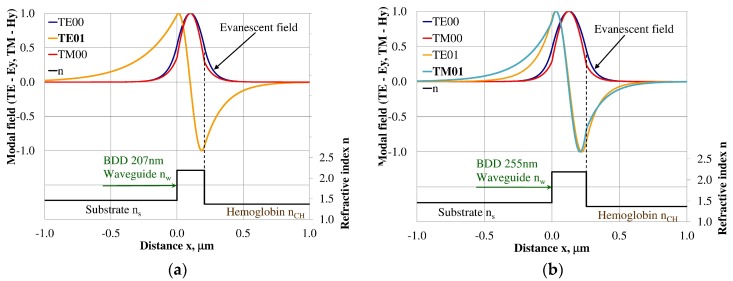
The modal field distribution in the multi-mode BDD-based waveguide: (**a**) TE01 for maximum *dN_eff_/dn_cH_*, and (**b**) TM01 for maximum *dN_eff_/dn_cH_*.

**Figure 10:**
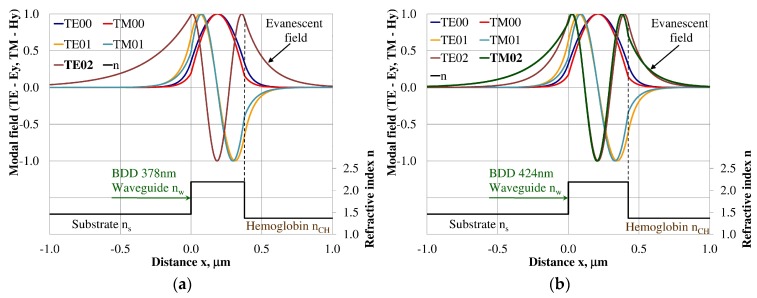
The modal field distribution in the multi- mode BDD-based waveguide: (**a**) TE02 for maximum *dN_eff_/dn_cH_*, and (**b**) TM02 for maximum *dN_eff_/dn_cH_*.

Design of an integrated optics sensor structure based on evanescent field phenomena of a guided waveguide mode requires analysis of the modal field distribution. The distribution of the modal field in a planar waveguide structure is a function of the refractive index of the waveguide layer *n_w_*, its thickness *d_w_*, and the refractive indices of the substrate *n_s_*, cover layer *n_cH_*, and cladding *n_c_* layer, the wavelength λ, and polarization of light. The modal field distribution was analyzed in the NDD- and BDD-based waveguide structures covered with a hemoglobin layer, where the thickness of the waveguide layer thickness was assumed to correspond to the maximum homogeneous sensitivity *dN_eff_/dn_cH_* presented in Table 3. The modal field distributions that can propagate into the waveguide structure corresponding to the maximum homogeneous sensitivity are presented in Figure 5, Figure 6, Figure 7, Figure 8, Figure 9 and Figure 10 (marked in bold). Other modal fields which can propagate into a waveguide structure as guided modes are also shown for comparison purposes. The evanescent field of a guided mode penetrates the cover layer and substrate. Changes in the refractive index or extinction coefficient of the cover layer directly influence the propagation conditions for waveguide modes, i.e., the effective refractive index and attenuation coefficient of light. These two effects can be used in sensors structures for determining the optical properties of the cover layer (in this case, hemoglobin). Struk et al. in [10,11,12] described the operation of integrated optics sensor structures for measuring changes in the refractive index or extinction coefficient of the cover layer, which requires the use of a grating coupler or circuit with prism coupler. One should note that the optical properties of the substrate also influence the modal field distribution in the sensors’ structures. The evanescent field of guided modes penetrates the substrate layer, however, this phenomenon is irrelevant in sensor applications because the optical and geometric properties of the substrate are constant and they do not cause changes in the propagation conditions in the sensor. 

The modal field distributions of a guided mode with marked evanescent field into single-mode waveguides at the highest homogeneous sensitivity for the NDD-based or BDD-based waveguide structures are shown in Figure 5a,b and Figure 8a,b, respectively. In this case, a large portion of the modal field for TE00 or TM00 modes penetrates the cover layer. In the multi-mode waveguide, the penetration depth of the evanescent field is larger for higher-order modes compared to the lower-order modes. The modal field for the lower-order modes is more focused in the waveguide layer, therefore it penetrates the surrounding layers to a lesser extent. The modal field distribution and evanescent field in the multi-mode NDD-based or BDD-based waveguides are shown in Figure 6a,b and Figure 7a,b (TE0, TE1, TE01, TM0, TM01, and TM02) and Figure 9a,b and Figure 10a,b (TE0, TE1, TE01, TM01, TM01, and TM02), respectively.

### 3.4. Propagation of Modes in Planar Waveguide Structure with Hemoglobin Cover Layer

The waveguide modes during propagation in the planar waveguide structure with length *L* scan the cover layer by evanescence field. The waveguide mode is attenuated in the waveguide structure if an imaginary part of refractive index *k* is different from 0. Analysis of the modes propagation in waveguide structure were completed with the assumption that the imaginary part of the refractive index for oxidized hemoglobin and non-oxidized hemoglobin is: k_HbO2_436_ = 0.0023, k_Hb_436_ = 0.0095 for *λ* = 436 nm, and k_HbO2_560_ = 0.00073, k_Hb_560_ = 0.0012 for *λ* = 560 nm. The imaginary parts of refractive indices *k* were calculated based on molar extinction coefficient for oxidized and non-oxidized hemoglobin in water presented by Prahl [24]. The calculated imaginary part of refractive index *k* is well correlated with measured data presented by Sydoruk et al. [25]. The propagation of the waveguide modes are presented for selected cases: the NDD-based structure with waveguide layer thickness *d_w_* corresponds to maximum homogenous sensitivity for single-mode or multi-mode waveguide structure, wavelength of light corresponds to the maximum difference of imaginary part of refractive index *k* between oxidized and non-oxidized hemoglobin (*λ* = 436 nm and *λ* = 560 nm). The sensitivity of hemoglobin oxidation level detection has been calculated based on equation:

(7) $$S_{H}\;=\;10\;\log\;\frac{P_{\mathrm{Hb}}}{P_{\mathrm{HbO}2}}$$

where: P_Hb_—transmitted power with non-oxidized hemoglobin cover layer, P_HbO2_—transmitted power with oxidized hemoglobin cover layer, *L*—propagation distance *L* = 100 μm. 

**Figure 11:**
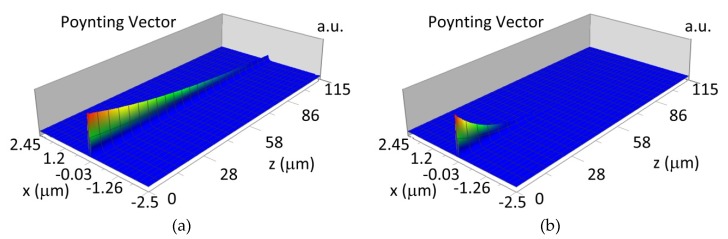

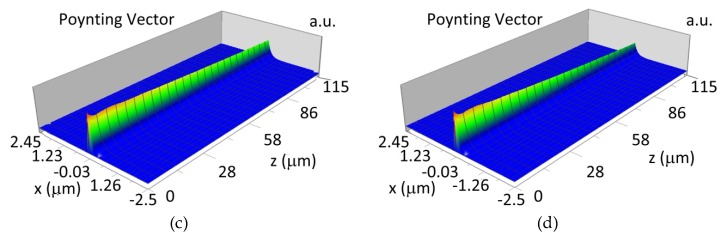
Propagation light in single-mode waveguide structure for TE00, *d_w_* = 32 nm (Poynting vector): (**a**) *λ* = 436 nm, HbO_2_, (**b**) *λ* = 436 nm, Hb, (**c**) *λ* = 560 nm, HbO_2_, (**d**) *λ* = 560 nm, Hb.

**Figure 12:**
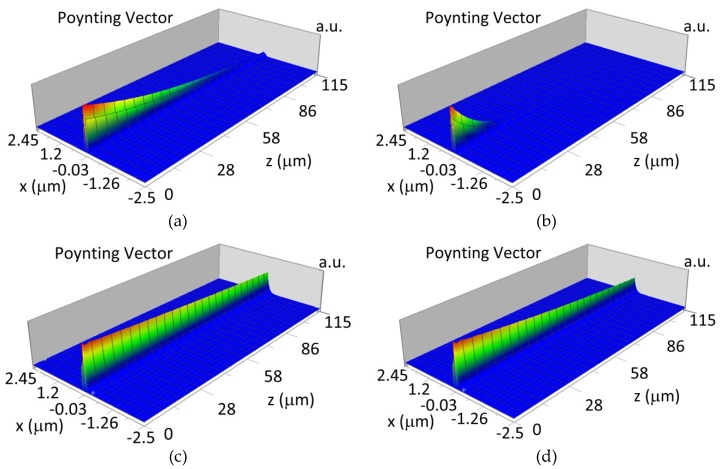
Propagation light in single-mode waveguide structure for TM00, *d_w_* = 85 nm (Poynting vector): (**a**) *λ* = 436 nm, HbO_2_, (**b**) *λ* = 436 nm, Hb, (**c**) *λ* = 560 nm, Hb_O2_, (**d**) *λ* = 560 nm, Hb.

**Figure 13:**
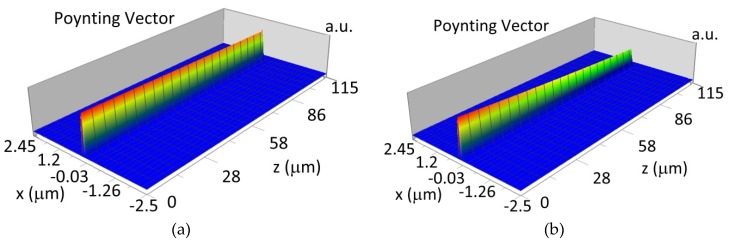

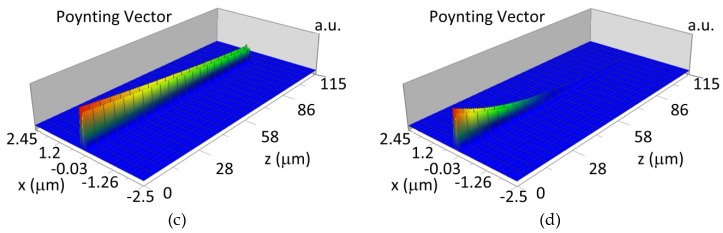
Propagation light in multi-mode waveguide structure for *d_w_* = 188 nm, *λ* = 436 nm (Poynting vector): (**a**) TE00, HbO_2_, (**b**) TE00, Hb, (**c**) TE01, HbO_2_, (**d**) TE01, Hb.

**Figure 14:**
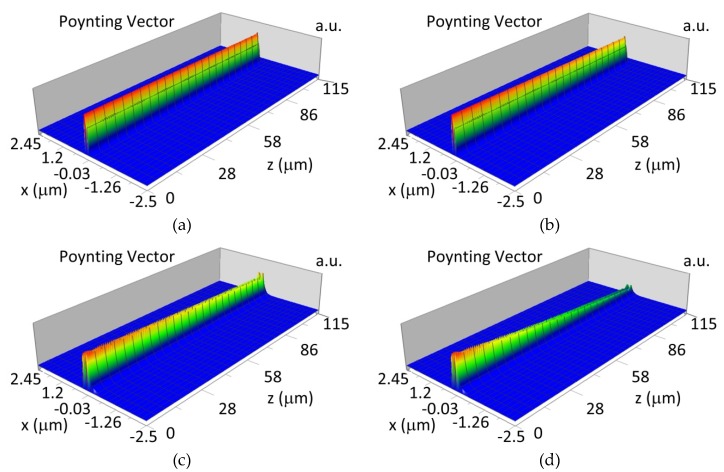
Propagation light in multi-mode waveguide structure for *d_w_* = 188 nm, *λ* = 560 nm (Poynting vector): (**a**) TE00, HbO_2_, (**b**) TE00, Hb, (**c**) TE01, HbO_2_, (**d**) TE01, Hb.

The propagation of TE00 modes in single-mode waveguide (*d_w_* = 32 nm) with oxidized hemoglobin (HbO_2_) or non-oxidized hemoglobin (Hb) cover layer for wavelength *λ* = 436 nm is presented in Figure 11a,b and *λ* = 560 nm are presented in Figure 11c,d The calculated TE00 sensitivity of detection of hemoglobin oxidation level is S_H436_ = 21 dB for wavelength *λ* = 436 nm or S_H560_ = 1.2 dB for wavelength *λ* = 560 nm. The propagation of TM00 modes in single-mode waveguide (*d_w_* = 85 nm) with oxidized (HbO_2_) or non-oxidized (Hb) hemoglobin cover layer for wavelength *λ* = 436 nm are presented in Figure 12a,b and for *λ* = 560 nm in Figure 12c,d. The calculated sensitivity S_H_ of hemoglobin oxidation level detection is S_H436_~25 dB for wavelength *λ* = 436 nm or S_H560_ = 1.3 dB for wavelength *λ* = 560 nm (TM00 mode). The propagation of TE00, TE01 modes in multi-mode waveguide (*d_w_* = 188 nm) with oxidized or non-oxidized hemoglobin cover layer are presented in Figure 13a–d (wavelength *λ* = 436 nm) and Figure 14a–d (wavelength *λ* = 560 nm). The sensitivity of the hemoglobin oxidation level detection for following modes is: TE00 S_H436_ = 1.6 dB, TE01 S_H436_ = 9 dB, TM00 S_H436_ = 4.3 dB for wavelength *λ* = 436 nm and for TE00 S_H560_ = 0.4 dB, TE01 S_H560_ = 2.7 dB for wavelength *λ* = 560 nm, TM00 S_H436_ = 0.4 dB. 

**Figure 15:**
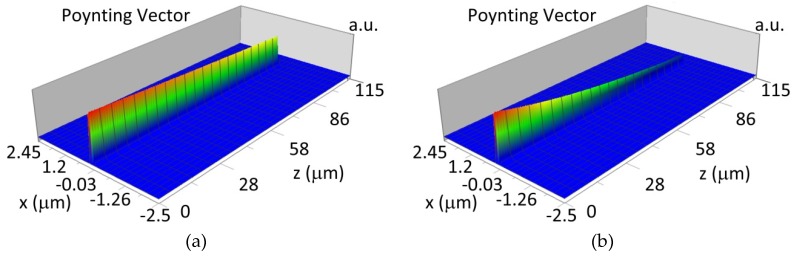

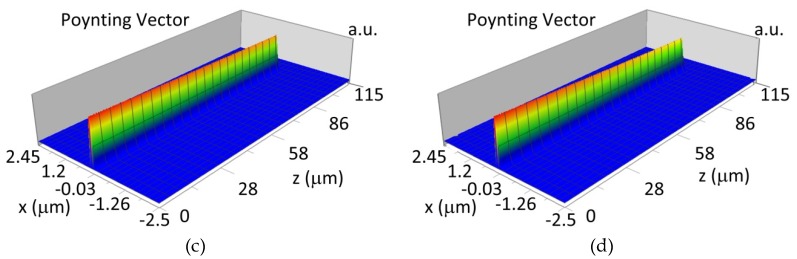
Propagation light in waveguide structure for TM00, *d_w_* = 188 nm (Poynting vector): (**a**) *λ* = 436 nm, HbO_2_, (**b**) *λ* = 436 nm, Hb, (**c**) *λ* = 560 nm, Hb_O2_, (**d**) *λ* = 560 nm, Hb.

**Figure 16:**
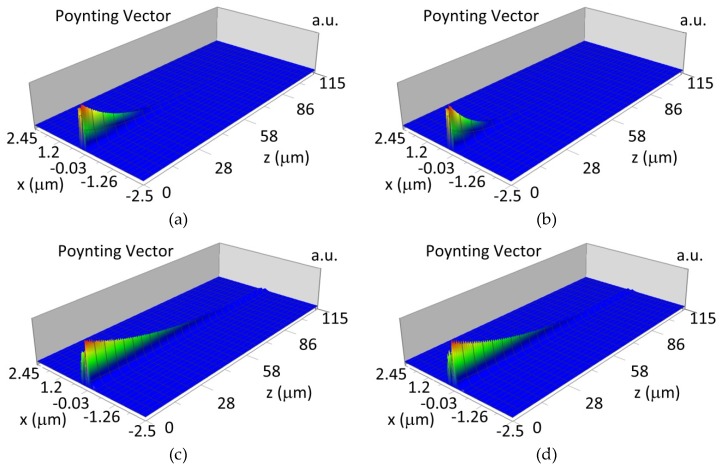
Propagation light in waveguide structure for TM01, *d_w_* = 237 nm (Poynting vector): (**a**) *λ* = 436 nm, HbO_2_, (**b**) *λ* = 436 nm, Hb, (**c**) *λ* = 560 nm, HbO_2_, (**d**) *λ* = 560 nm, Hb.

The propagation of TM00 mode in the multi-mode waveguide structure (*d_w_* = 188) with oxidized or non-oxidized hemoglobin cover layer is presented in Figure 15a,b (wavelength *λ* = 436nm) and Figure 15c,d (wavelength *λ* = 560 nm). The propagation of modes TM01 in multimode waveguide (*d_w_* = 237 nm) with oxidized or non-oxidized hemoglobin cover layer is presented in Figure 16a,d, the sensitivity is S_H436_ = 14 dB for wavelength 436 nm and Figure 16c,d for the wavelength 560 nm sensitivity is S_H560_ = 1 dB. 

The calculated sensitivity of hemoglobin oxidation level detection S_H_ is dependent on evanescence field (area) which penetrate the cover layer and on optical properties of cover layer. The highest sensitivity S_H_ is for waveguide mode in single-mode planar waveguide structure and for wavelength *λ* = 436 nm. However, for wavelength *λ* = 436 nm, the modes are highly attenuated in waveguide structure, which means the waveguide length (distance between prism coupler and grating coupler) should be short at the level of ~25 μm. In addition, coupling of light into a waveguide structure by a prism coupler and uncoupled to cladding by grating coupler could be difficult to perform for this wavelength. In addition, fabrication of a single-mode diamond-based waveguide layer with a thickness of a few dozen nanometers and with suitable optical properties could be difficult.

The multi-mode diamond-based waveguide structure with a waveguide layer thickness at the level of a few hundred nanometers, operating for a wavelength *λ* = 560 nm could be also used for determination of hemoglobin properties (real part *n* and imagine part *k* of refractive index). The sensitivity S_H_ and changes of angle ∆*α* as a function of hemoglobin properties are a little bit lower in comparison to single-mode waveguide, however, the coupling and uncoupling of light by a prism and grating coupler will be easier, in addition fabrication technology of grating coupler, and diamond-based waveguide layer with thickness of a few hundred nanometers could be much more easier. 

## 4. Conclusions

This paper presents the concept and theoretical analysis of a diamond-based integrated optics structure for sensor applications and research on the properties of a hemoglobin concentration and hemoglobin oxidation level (real part *n* and imaginary part *k* of refractive index). The presented theoretical analysis facilitates a comparison of waveguide properties and sensitivity for two types of sensor structures based on NDD or BDD waveguide layers. The modal characteristics presented in the first part of the manuscript show the NDD-based waveguide has a higher effective refractive index than the BDD-based waveguide. In addition, the critical thickness of the waveguide layer, which is necessary for waveguide modes propagation, is lower in the case of NDD-based waveguide than the BDD-based waveguide. The critical thickness of the NDD-based waveguide layer can be smaller compared to the BDD-based waveguide by a dozen nanometers for a single-mode waveguide, or up to several tens of nm in the case of a multi-mode waveguide. 

The thickness of the waveguide layer is important for choosing the appropriate deposition technology and optimizing the deposition process. An analysis of the sensitivity confirms that the NDD-based waveguide sensor has higher homogeneous sensitivity than the BDD-based waveguide sensor. One should note that the homogeneous sensitivity is greatest for the zero-order mode in the single-mode waveguide, and the homogeneous sensitivity decreases for higher order modes in the multi-mode structure. The homogeneous sensitivity is also dependent on the polarization of the guided mode; the homogeneous sensitivity is higher for TM modes and lower for TE modes. The highest homogeneous sensitivity was observed for the structure with the highest refractive index contrast between the waveguide layer and the substrate and cladding. 

The presented numerical analysis confirms the high sensitivity of the proposed integrated optics sensor structure for research of optical properties: refractive index *n* of cover layer, and hemoglobin by grating coupler and high sensitivity for research of imaginary part of refractive index *k* of hemoglobin by evanescence field of guided waveguide mode.

In summary, the theoretical analysis shows the optimal geometrical and optical properties of an integrated optics-based sensor structure with diamond waveguide, prism coupler and grating coupler for research into the optical properties of a hemoglobin cover layer (hemoglobin concentration and hemoglobin oxidation).

## Figures and Tables

**Table 1:** The refractive index for each layer in modeled waveguide structure.

Wavelength (nm)	Refractive Index				
	Non-Doped Diamond *n_wNDD_* [21]	Boron-Doped Diamond *n_wBDD_* [21]	Quartz Substrate *n_s_*	Air	Biological liquid-Hemoglobin *n_cH_* [23] * 0/17.3/26.0 g/dL
560	2.30	2.19	1.4595	1.0003	1.3342/1.3681/1.3836

* The proper level of hemoglobin level in human blood is 12–18 g/dL (depending of age and sex) [28]. The refractive index of hemoglobin is based on available data in literature (*λ* = 546 nm) [23].

**Table 2:** The critical thickness of waveguide layer for waveguide modes.

Structure	Waveguide Layer Thickness *d_w_*, nm					
	Single-Mode		Multi-Mode			
	TE00	TM00	TE01	TM01	TE02	TM03
NDD/Quartz	31	69	186	224	343	380
Hemoglobin/NDD/Quartz	19	42	172	194	329	351
BDD/Quartz	35	75	204	242	375	413
Hemoglobin/BDD/Quartz	22	45	189	211	360	382

**Table 3:** Analysis of maximum homogeneous sensitivity *d_Neff_/dn_cH_*, changes of the angle Δ*α* as a function of different hemoglobin concentration (Figure 1).

**Waveguide Layer Thickness *d_w_* (nm) for Maximum Homogeneous Sensitivity *dN_eff_/dn_cH_***											
**Hemoglobin/NDD/Quartz**						**Hemoglobin/BDD/Quartz**					
**Single-mode**		**Multi-Mode**				**Single-mode**		**Multi-Mode**			
TE00	TM00	TE01	TM01	TE02	TM02	TE00	TM00	TE01	TM01	TE02	TM02
32	85	188	237	345	393	37	89	207	255	378	424
**Expected changes of the angle ∆*α*(°) as a function of hemoglobin concentration 0–26 g/dL.**											
0.95	1.28	0.65	0.94	0.51	0.77	0.91	1.19	0.62	0.87	0.48	0.71
